# Regulatory variant in *FZD6* gene contributes to nonsyndromic cleft lip and palate in an African-American family

**DOI:** 10.1002/mgg3.155

**Published:** 2015-05-07

**Authors:** Nevena Cvjetkovic, Lorena Maili, Katelyn S Weymouth, S Shahrukh Hashmi, John B Mulliken, Jacek Topczewski, Ariadne Letra, Qiuping Yuan, Susan H Blanton, Eric C Swindell, Jacqueline T Hecht

**Affiliations:** 1 Department of Pediatrics, University of Texas Medical School at HoustonHouston, Texas; 2 Graduate School of Biomedical Sciences, University of Texas Health Science CenterHouston, Texas; 3 Boston Children’s HospitalBoston, Massachusetts; 4 Department of Pediatrics, Northwestern University Feinberg School of Medicine, Ann & Robert H. Lurie Children’s Hospital of Chicago Research CenterChicago, Illinois; 5 University of Texas School of Dentistry at HoustonHouston, Texas; 6 Dr. John T. Macdonald Department of Human Genetics, Hussman Institute for Human Genomics, University of Miami Miller School of MedicineMiami, Florida

**Keywords:** Frizzled-6, nonsyndromic cleft lip and palate, regulatory variant, WNT pathway

## Abstract

Nonsyndromic cleft lip with or without cleft palate (NSCLP) is a common birth defect affecting 135,000 newborns worldwide each year. While a multifactorial etiology has been suggested as the cause, despite decades of research, the genetic underpinnings of NSCLP remain largely unexplained. In our previous genome-wide linkage study of a large NSCLP African-American family, we identified a candidate locus at 8q21.3-24.12 (LOD = 2.98). This region contained four genes, Frizzled-6 (*FZD6*), Matrilin-2 (*MATN2*), Odd-skipped related 2 (*OSR2*) and Solute Carrier Family 25, Member 32 (*SLC25A32*). *FZD6* was located under the maximum linkage peak. In this study, we sequenced the coding and noncoding regions of these genes in two affected family members, and identified a rare variant in intron 1 of *FZD6* (rs138557689; c.-153 + 432A>C). The variant C allele segregated with NSCLP in this family, through affected and unaffected individuals, and was found in one other NSCLP African-American family. Functional assays showed that this allele creates an allele-specific protein-binding site and decreases promoter activity. We also observed that loss and gain of *fzd6* in zebrafish contributes to craniofacial anomalies. *FZD6* regulates the WNT signaling pathway, which is involved in craniofacial development, including midfacial formation and upper labial fusion. We hypothesize, therefore, that alteration in *FZD6* expression contributes to NSCLP in this family by perturbing the WNT signaling pathway.

## Introduction

Isolated or nonsyndromic cleft lip with or without cleft palate (NSCLP) is the fourth most common birth defect in the United States (MMWR 2006; Hashmi et al. 2005; Parker et al. 2010). Despite improvement in treatment, NSCLP continues to have major medical, psychosocial, and financial burdens for the affected individuals and their families (Strauss 2001; Mossey and Castilla 2003; Christensen et al. 2004; Wehby and Cassell 2010; Demir et al. 2011). Therefore, identification of the causes of this craniofacial anomaly is of critical importance.

There is considerable variation in the birth prevalence among ethnic groups, with NSCLP occurring most commonly in Asians and Native Americans (1/500), followed by non-Hispanic Whites (NHW) and Hispanic populations (1/700–1/1000), and least commonly in African-Americans (1/2500) (Spritz 2001; Wyszynski 2002b; Hennekam et al. 2010). Estimates are even lower for most regions of the African continent (1/3300) (Butali and Mossey 2009). This suggests differences in genetic liability consistent with a multifactorial etiology (Wyszynski 2002). Indeed, this mechanism of multiple genetic and environmental interactions was first proposed for NSCLP more than 40 years ago (Carter 1969, 1976). Evidence for a genetic etiology is based on higher concordance in monozygotic (25–40%) than dizygotic (3–6%) twins, aggregation within families with a 10- to 32-fold increase in recurrence risk for first-degree relatives and a heritability of 76% (Hecht 1990; Mitchell and Risch 1992; Christensen and Fogh-Andersen 1993; Wyszynski et al. 1996; Wyszynski 2002; Lidral and Murray 2004; Sivertsen et al. 2008; Grosen et al. 2010; Hennekam et al. 2010). Numerous approaches have been used to identify the underlying molecular causes resulting in the characterization of 12–20% of the genetic variation. Thus, most of the genetic variation underlying NSCLP is unexplained (Wyszynski 2002; Lidral and Murray 2004; Lidral and Moreno 2005; Mossey et al. 2009; Dixon et al. 2011; Stuppia et al. 2011).

**Figure 1:**
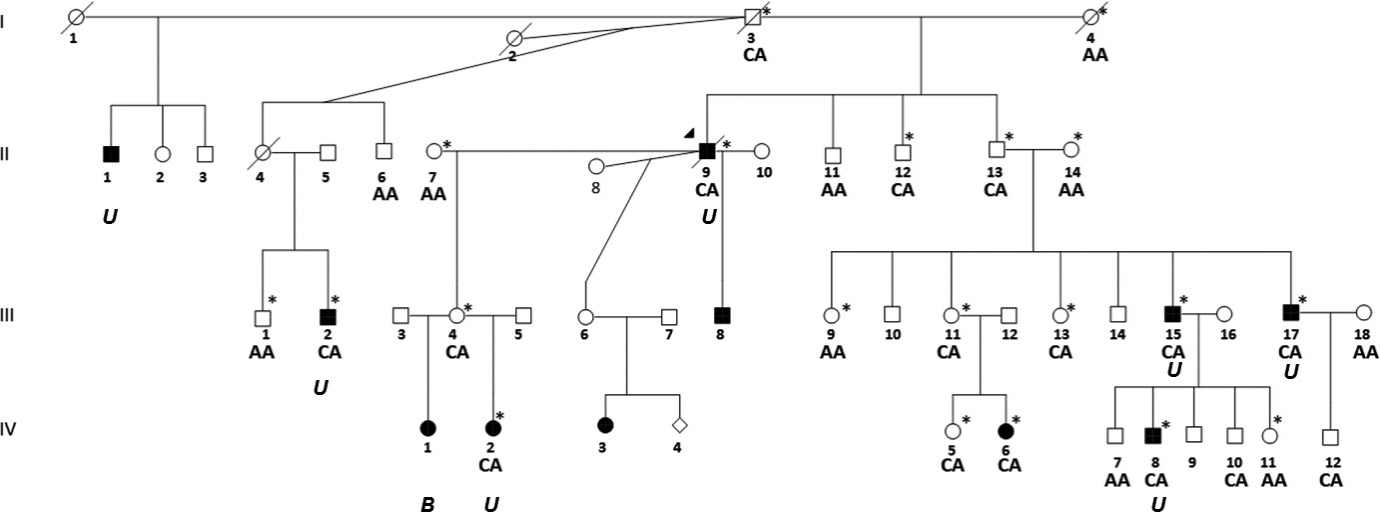
Multiplex NSCLP African-American family. Pedigree depicts eleven individuals with NSCLP spanning three generations. Filled symbols denote affected individuals and asterisks (*) denote individuals included in the genome scan reported by Chiquet et al. (2009). Laterality is indicated as B for bilateral and U for unilateral. All of the examined individuals had cleft lip with cleft palate. C allele of rs138557689 segregates with affected individuals and is transmitted by four unaffected individuals originating from individual I-3. C allele is also present in 5 additional unaffected individuals.

As part of our NSCLP gene studies, we ascertained a large multiplex African-American family with 11 cases of NSCLP segregating in 3 generations (Fig.1). This family is unique because NSCLP is less common in African-American populations. We previously reported suggestive evidence for linkage in this family under a dominant model with reduced penetrance (penetrances of 0.24 and 0.32 in females and males, respectively; phenocopy rate of 0.001) (Chiquet et al. 2009) to the 8q21.3-24.12 region (LOD = 2.98, maximum possible LOD) flanked by rs150615 and rs2034844 (chr8: 90638305-121,344,027). This was the only region shared by all affected individuals. In other unpublished studies, there was no evidence for structural variation segregating with the phenotype in the family. A meta-analysis of 13 NSCLP genome scans previously reported association to the 8q24.21 region (Marazita et al. 2002). The 8q24.21-associated region is defined by rs3857888-rs17821251 (chr8:129894043-130078415) and does not overlap with our linkage region (8 Mb away). Inspection of the linkage region identified four candidate genes: Frizzled-6 (*FZD6*), Matrilin-2 (*MATN2*)*,* Odd-Skipped Related 2 (*OSR2*), and Solute Carrier Family 25 Member 32 (*SLC25A32*). Herein, we report the identification of a rare intronic variant in *FZD6* that segregates with the phenotype and creates a novel allele-specific protein-binding site that decreases promoter activity.

## Materials and Methods

### IRB approval

This study was approved by the Committee for the Protection of Human Subjects at the University of Texas Health Science Center at Houston (HSC-MS-03-090/HSC-MS-11-0336).

### NSCLP multiplex family

An African-American NSCLP family with 24 individuals, 11 affected with NSCLP (7 of whom were available for evaluation) and 13 unaffected individuals, was ascertained (Fig.1). All family members from whom blood was obtained underwent clinical examination to exclude known syndromic causes of orofacial clefting by one author (JTH). All affected individuals had cleft lip with cleft palate and the laterality information where known is indicated on pedigree. No other anomalies, including lip pits, were present in any family members. DNA samples from the 7 affected and 13 unaffected family members were subjected to a 6K Illumina (San Diego, CA) IVb genome scan and linkage analysis as previously described (Chiquet et al. 2009).

### Targeted sequencing

NCBI GenBank (www.ncbi.nlm.nih.gov) was used to determine the genomic structure of *FZD6* (RefSeq NM_00164615.1), *MATN2* (NM_002380.3)*, OSR2* (NM_001142462.2), and *SLC25A32* (NM_030780.3). Forward and reverse primers were designed to capture each exon and approximately 50–100 bps upstream and downstream of the intron/exon junction, as well as the complete 5′ and 3′ untranslated regions (UTRs) for all three isoforms of *FZD6*, two isoforms of *MATN2, OSR2,* and *SLC25A32* (Tables S1–S3). DNA samples from two affected family members (Fig1: III-15 and IV-2) were sequenced for each gene. Standard PCR amplification conditions were used and the annealing temperatures for each primer set are shown in the Tables S1–S3. Amplified PCR products were purified according to manufacturer’s protocol (Qiagen, Valencia, CA). Sequencing results were compared to consensus sequences obtained from NCBI public database and analyzed using Sequencer v4.9 (Gene Codes, Ann Arbor, MI). The *FZD6* variant rs138557689 was sequenced in 5 affected and 19 additional family members using primer set E1C (Table S1).

### Variant analyses

Only potentially functional sequence changes shared by both affected individuals (Fig1: III-15 and IV-2) were considered (www.ncbi.nlm.nih.gov/projects/SNP). SNPs identified in the potential regulatory regions, 5′ UTR and the first two introns of the gene, were assessed for their effect on protein binding using three in silico functional prediction programs: Alibaba2, Patch, and Transcription Element Search Software (TESS) (Grabe 2002; Matys et al. 2006; Schug 2008). SNPs identified in the 3′UTR region were assessed for their effect on microRNA binding using microRNAMap and miRBAse databases (Griffiths-Jones et al. 2008; Hsu et al. 2008). SNPs found in the coding region were analyzed using PolyPhen and SIFT (Ng and Henikoff 2001; Adzhubei et al. 2010).

### Species conservation analysis

SNPs were assessed for evolutionary conservation using the UCSC Genome Browser Multi Alignments and Conservation (www.genome.ucsc.edu/cgi-bin/hgGateway) and the ECR Browser tool (http://ecrbrowser.dcode.org). Genomic Evolutionary Rate Profiling (GERP) score within the UCSC browser was used to estimate the evolutionary constraint rates for individual nucleotide positions (Cooper et al. 2005).

### Replication and frequency analyses

The *FZD6* rs138557689 variant was genotyped in an additional 836 unrelated individuals with NSCLP and 579 control individuals (Table S4). A custom TaqMan Genotyping Assay (Applied Biosystems, Foster City, CA) was designed using standard protocols and run on a ViiA7 Sequence Detection Instrument (Applied Biosystems). Allele calls were determined using the ViiA7 EDS software (Applied Biosystems). Allele call rates were greater than 98%.

### Whole exome sequencing

The 8q21.3-24.12 region spans approximately 35.6 Mb and contains numerous genes. To identify variants in other genes under the linkage peak, we performed whole exome sequencing on all available affected and connecting family members. The SureSelect Human All Exon 50 Mb + UTRs kit (Agilent, Santa Clara, CA) was used for in-solution enrichment of coding exons and flanking intronic sequences following the manufacturer’s standard protocol. Adapter sequences for the Illumina HiSeq2000 were ligated and the enriched DNA samples were prepared using the standard methods for the HiSeq2000 instrument (Illumina). Paired-end reads of 99 bases length were produced. The Illumina CASAVA v1.8 pipeline was used to assemble 99 bp sequence reads. BWA (Li and Durbin 2009) was used to align sequence reads to the human reference genome (hg19) and variants were called using the GATK software package (McKenna et al. 2010; DePristo et al. 2011). All variants were submitted to SeattleSeq134 to assess functional consequences.

### Electrophoretic mobility shift assay

Gene sequence for *FZD6* was obtained from NCBI and two oligonucleotide probes were designed to incorporate approximately 10 bp upstream and downstream from rs138557689. Oligonucleotides were synthesized by Integrated DNA Technologies (Coralville, IA), annealed and end-labeled with P^32^ dCTP (Perkin Elmer, Waltham, MA). Cos-7 cell nuclear extracts were purchased from ActiveMotif (Carlsbad, CA). Electrophoretic mobility shift assy (EMSAs) were performed by incubating 2.5 *μ*g nuclear extract, radiolabeled probe and 1 *μ*L of dG/dC in a 20 *μ*L mixture containing 20 mmol/L Tris pH 7.5, 50 mmol/L KCl, 10% glycerol, 0.5 mmol/L EDTA (ethylenediaminetetracetic acid), 0.5 mmol/L DTT (dithiothreitol), 0.05% NP-40 (nonyl phenoxypolyethoxylethanol) and 1 mmol/L PMSF (phenylmethanesulfonylfluoride) for 1 h at 4°C. Competition assays included 5-, 10-, and 50-fold excess of cold probe. Negative controls were prepared using the labeled probes and binding buffer without the nuclear extract. All samples were loaded on a prerun 5% polyacrylamide gel in 1× TBE. After electrophoresis for 3 h at 150 V, the gel was dried and exposed to radiographic film at −80°C for 18–48 h, after which the film was developed.

### Generation of promoter constructs and luciferase reporter assay

The *FZD6* putative promoter sequence was identified using information from GeneCopeia (Rockville, MD) and SwitchGear Genomics (Menlo Park, CA) and included the 2277 bp region upstream of the transcriptional start site. This region was amplified from a BAC RP11-7N7 containing *FZD6* using forward primer ctagcccgggctcgagCATCAGTAAGCTTTTGATAA and reverse primer cttagatcgcagatctTTTCTTCAAATTCCTGATTTTACC following standard PCR conditions with the addition of betaine. PCR primers were designed to incorporate 5′-Xho I and 3′- Bgl II cut sites for ligation into the double digested pGL4.10 luciferase basic vector (pGL4.10, Promega, Madison, WI) using the In-Fusion® HD Cloning System (Clontech, Mountain View, CA). The insert was sequenced and aligned to the NCBI consensus sequence (http://www.ncbi.nlm.nih.gov). The *FZD6* alternate rs138557689/C allele was created using site-specific mutagenesis (QuikChange® II kit, Agilent Technologies).

For transfection of each *FZD6* construct, Cos-7 cells were first seeded at 200,000 cells/well for 24 h. The X-tremeGENE (Roche, Indianapolis, IN) reagent was used to transfect 2.0 *μ*g of luciferase reporter construct and 0.05 *μ*g of *Renilla* internal control. Transfection was performed in Hek293T cells using 100,000 cells/well and FugeneHD (Promega). All experiments were performed in triplicate and repeated at least three times. Luciferase activities were measured 48 h after transfection using the dual-luciferase system in a Glomax 20/20 luminometer (Promega). Luciferase activity was normalized to the activity of the Renilla internal control to correct for variation in transfection efficiencies. Unpaired *t*-tests were used to compare luciferase expression between ancestral and alternate constructs. *P* ≤ 0.05 were considered statistically significant.

### Zebrafish assays

Zebrafish (*Danio rerio*) were raised and housed following standard techniques (Westerfield 1993).

#### Morpholino (MO)/mRNA injections

Two nonoverlapping zebrafish *fzd6* antisense start site MOs (MO1: TTAACCGCAAACCTCCTCCTCTTCC, MO2: TCCTCCAGAAACGGAATGTCGCTCA) and one mismatch MO (MM: TCCTCGACAAACCGAATCTCCCTCA) were obtained from GeneTools (Philomath, OR) and optimized. MOs were diluted in nuclease-free water to a stock concentration of 65 mg/mL or 2 mmol/L, and further diluted to a working concentration of 12 mg/mL. Injections (0.5 mg/mL) were diluted in Danieu buffer and 0.5 *μ*L of 2% phenol red was added to facilitate injections. One-cell embryos were injected with 1 nL of MO and incubated at 28°C. Embryos were observed during development up to 6 days postfertilization (dpf) and collected at 24 h and 6 dpf. A full-length zebrafish *fzd6* cDNA was cloned into a pCS2 vector and *fzd6* mRNA was generated using the mMessage mMachine kit (Life Technologies, Carlsbad, CA). 1 nL of mRNA was injected at a concentration of 25 ng/*μ*L. Injection volume was calculated by measuring the diameter of injected droplet.

#### Cartilage/bone staining and imaging

Zebrafish embryos were collected at 6 dpf for cartilage and bone staining using standard techniques (Sprague et al. 2006). Imaging was performed using the LAS Montage Module (Leica, Wetzlar, Germany).

## Results

Sequencing the coding and 5′ and 3′ UTR regions for *FZD6, MATN2, OSR2,* and *SLC25A32* in two affected individuals, III-15 and IV-2, identified three shared sequence variants. rs11345830, located in the 3′UTR of *SLC25A32,* is a homozygous deletion of nucleotide T (c.*164delT) (www.ncbi.nlm.nih.gov/projects/SNP). While highly conserved among primates, this homozygous deletion was not predicted to affect microRNA-binding sites and was excluded from further analysis. A second variant, rs113199627, identified in the 5′UTR of *MATN2*, is common in the population (MAF = 14.5%) and was also excluded from further analysis. No shared variants were identified in *OSR2*. WES identified three potentially pathogenic variants in this region; however, none of these were present in all affected individuals in this family. Moreover, none of the potentially pathogenic variants outside of the linked region were shared by all affected individuals.

A single base pair variant in *FZD6*, rs138557689/C (c.-153 + 432A>C), was present in both affected individuals. rs138557689 is located in intron 1, 681bps upstream of the transcriptional start site in exon 2. Based on availability of DNA samples, 24 additional relatives were genotyped. The C allele was present in all affected individuals and was transmitted by four unaffected individuals (Fig.1: I-3, II-13, III-4, III-11). There were five other unaffected family members with the CA genotype (Fig.1: II-12, III-13, IV-5, IV-10, IV-12).

This variant is reported in 1000 Genomes with an allele frequency of <1% in African-Americans and 0% in other populations (www.1000genomes.org/). In addition, we genotyped an independent case–control data set composed of 836 NSCLP probands (459 NHWs, 292 Hispanics, and 85 African-Americans) and 579 controls (150 NHWs, 304 Hispanics, and 125 African-Americans). The rs138557689/C allele was found in three controls (1 NHW and 2 African-Americans), all of whom were heterozygous. No homozygous CC individuals were identified. Among controls, the frequency of the rs138557689/C allele was 0.8% in African-Americans, 0.3% in NHWs and 0% in Hispanics (Table1). Among cases, we identified one African-American proband who was heterozygous for rs138557689, which he inherited from his unaffected father. The C allele was not found in any NHW or Hispanic NSCLP probands (Table1). Among affected probands, the frequency of the rs138557689/C allele was 1.2% for African-Americans and 0% for the NHW and Hispanics (Table1). Thus, these data suggest that rs138557689/C is a rare variant.

**Table 1:** Frequency of rs138557689A and C alleles

Genotypes	NHW	African-American	Hispanic
(A) Controls			
A	299	248	608
C	1	2	0
C allele frequency (%)	0.3	0.8	0.0
(B) NSCLP probands			
A	918	170	584
C	0	2	0
C allele frequency (%)	0.0	1.2	0.0

NHW, Non-Hispanic White; A-A, African American; Hisp, Hispanic.

To assess the conservation of the sequence, we compared the allelic sequences in different species. The ancestral rs138557689/A allele showed conservation in chimpanzees, rhesus monkeys, gorillas, marmosets, mouse lemurs, and opossums, while the alternate rs138557689/C allele was only conserved in elephants and armadillos (Fig. S2). The location of rs138557689 did not show evidence of evolutionary constraint (GERP score = -2.98). This is not surprising, as we hypothesize that this variant is only one of a number factors contributing to the phenotype.

**Figure 2:**
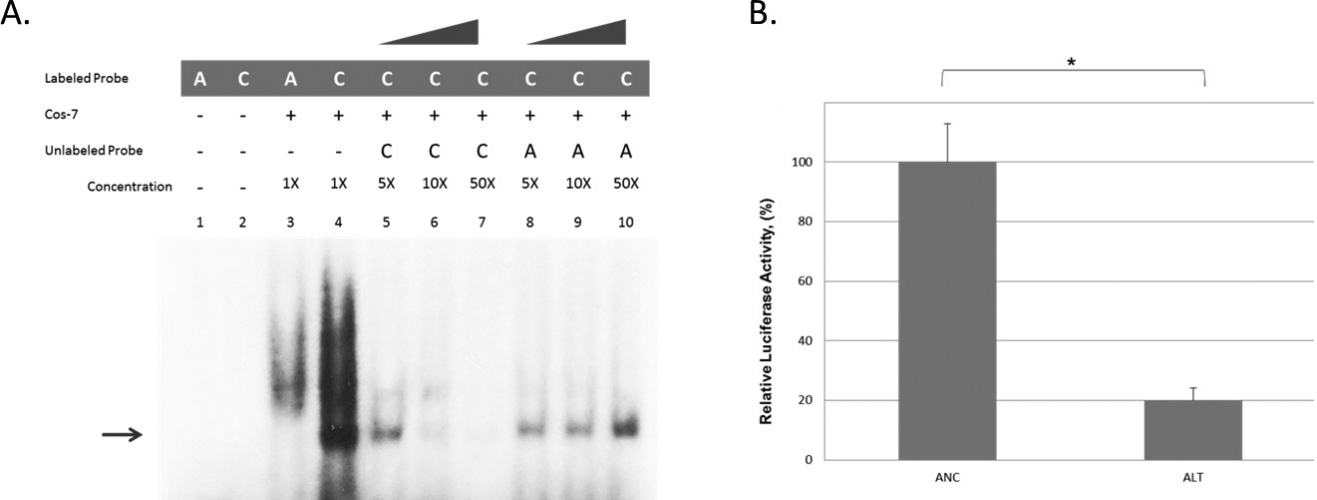
*FZD6* rs138557689/C creates an allele-specific protein-binding complex and decreases *FZD6* promoter activity. (A) Electrophoretic mobility shift assays (EMSA) were performed using nuclear extract from Cos7 cells. Samples were incubated with P^32^-labeled oligonucleotides containing either the ancestral A or alternate C alleles, or with unlabeled ancestral A or alternate C serving as specific competitors. Poly-dCdG was used as a nonspecific competitor. Negative controls were run using labeled probes without the nuclear extract. Bands were observed with the C allele only and the alternate band was competed out with C competitor only. (B) Hek293T cells (100,000 cells/well) were seeded for 24 h and cotransfected with ancestral or alternate promoter construct and *Renilla* reporter construct. Luciferase activities were normalized to the internal *Renilla* control. Data represent mean values ± SD from three independent experiments. Alternate C allele showed significant decrease in activity (*P < 0.001, unpaired *t*-test).

EMSA was used to determine whether a protein-binding site was created by either allele. As shown in Figure2, there was one band present for the probe containing the rs138557689/C allele that was not present for rs138557689/A probe. This suggested that the rs138557689/C allele produces an allele-specific protein-binding site that cannot be competed out by the ancestral (A) allele (Fig.2A). As shown in Figure2B, luciferase reporter activity of *FZD6* was reduced by approximately 80% (p < 0.001) with the rs138557689/C (alternate allele) promoter construct. Similar results were obtained for Cos7 cells (data not shown).

**Figure 3:**
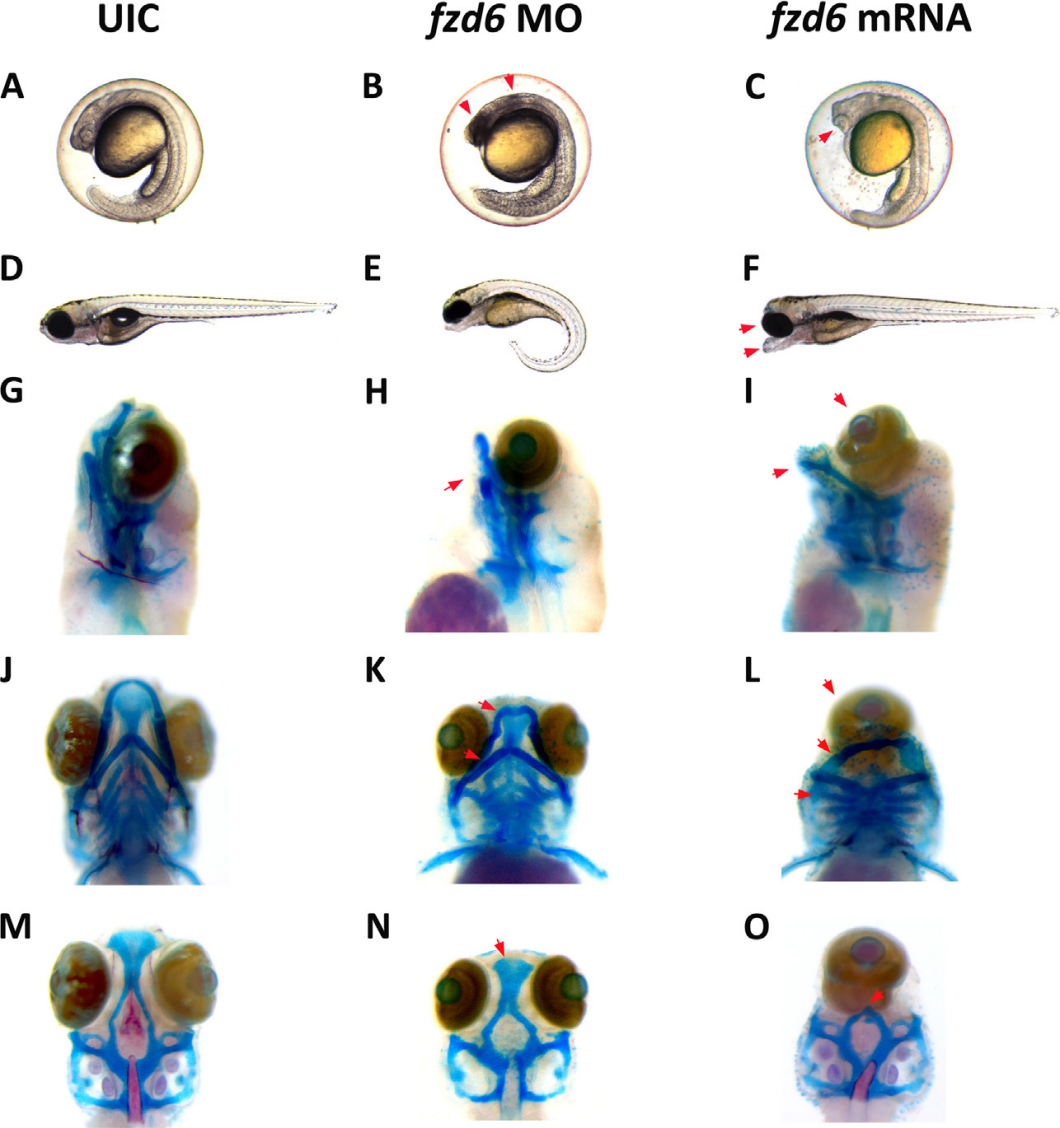
Knockdown and overexpression of FZD6 protein in zebrafish results in craniofacial defects. Both nonoverlapping morpholinos caused the same spectrum of craniofacial abnormalities. Only MO1-injected embryos are shown as an example. (A, D, G, J, and M) are uninjected control (UIC) embryos. (B, E, H, K, and N) show the phenotypes associated with knockdown of *fzd6*. (C, F, I, L, and O) show phenotypes associated with overexpression of *fzd6*. (A) UIC embryos at 24 hpf. (B) *fzd6* MO-injected embryos at 24 hpf. (C) *fzd6* mRNA-injected embryos at 24 hpf. (D) UIC embryos at 6 dpf. (E) *fzd6* MO-injected embryos at 6 dpf. (F) *fzd6* mRNA-injected embryos at 6 dpf. G-O show Alcian blue and alizarin red-stained embryos at 6 dpf. Jaw and palatal abnormalities due to fzd6 knockdown are shown (red arrows) in (K and N), respectively. Specifically in (K), arrows point to abnormal Meckel’s and ceratohyal cartilage in the lower jaw. In (N), arrows point to a reduced ethmoid palatal plate. Jaw and palatal abnormalities due to fzd6 overexpression are shown (red arrows) in (L and O), respectively. Specifically in (L), arrows point to abnormal Meckel’s and ceratohyal cartilage in the lower jaw. In (O), arrow points to a loss of the ethmoid plate in the palate.

To examine the role of *FZD6* during craniofacial development, two nonoverlapping antisense MOs directed against the translation start site of zebrafish *fzd6* were injected into one-cell embryos to knock down production of Fzd6 protein. Wild-type embryos injected with the nonoverlapping MOs resulted in necrosis at 24 hours postfertilization (hpf) (arrows in Fig.3A and B). By 6 dpf, *fzd6* MO-injected embryos exhibited abnormalities of craniofacial elements when compared to wild-type controls (Fig.3D and E). All *fzd6* MO-injected embryos developed abnormal Meckel’s and ceratohyal cartilage in the lower jaw (arrows in Fig.3H and K), and a reduced ethmoid plate (arrow in Fig.3N). No abnormalities were found upon injection of the mismatched MO. Full-length zebrafish *fzd6* mRNA was injected into one-cell embryos and allowed to develop to 6 dpf. Interestingly, overexpression of wild-type *fzd6* in zebrafish also resulted in a severe craniofacial phenotype with cyclopia when compared to wild-type controls (Fig.3C, F, I, L, and O). Compared to wild-type controls, *fzd6* mRNA-injected embryos develop severely abnormal Meckel’s and ceratohyal cartilage in the lower jaw (arrows in Fig.3I and L) and a complete loss of the ethmoid plate (arrow in Fig.3O).

## Discussion

Genome-wide linkage analysis, followed by targeted and whole-exome sequence analyses, were used to identify the genetic variation contributing to NSCLP in an African-American family with multiple affected members across three generations. Linkage was found to chromosomal region 8q21.3-24.12, which contains more than 100 genes (Fig. S1). Sanger sequencing of candidate genes led to the identification of rs138557689/C, a rare variant located 681 bp upstream of the transcription start site of *FZD6;* this variant segregated with NSCLP and significantly decreased promoter activity. The location of rs138557689 coincides with a region containing H3K27Ac (acetylation on histone 3 lysine 27 residue), a marker frequently present in enhancer regions and highly expressed in seven different cell types (https://genome.ucsc.edu), thus providing further evidence for a potential regulatory function for this variant. Moreover, rs138557689 is conserved, with a frequency of 0.8% in African-American controls and even less common in NHW and Hispanic controls (Table1). In addition to the original multiplex family, one other African-American NSCLP proband from a simplex family had the rs138557689/C variant suggesting that this may be a rare predisposing genetic cause of NSCLP. Nonetheless, the presence of the C allele in unaffected individuals in both families and in the controls clearly implies that other factors are critical to the expression of the clefting phenotype. Although we did not identify any other likely candidates from the linked or any other region in our WES, it is possible that the true causal variant was not captured by WES or it is not recognized by any of the prediction algorithms. However, the functional studies suggest that this variant is a contributing NSCLP factor.

NSCLP has long been considered to have a multifactorial etiology with contributions from multiple genes and environmental factors (Carter 1969, 1976). As rs138557689/C was found in both affected and unaffected individuals, it is clearly not sufficient to cause NSCLP. More likely, rs138557689/C acts in combination with variants in one or more genes and/or environmental factors to cause the phenotype. This leads us to hypothesize that the underlying risk profile varies between individuals in this family. Notably, this variant was present only in our African-American cases, supporting the notion that the underlying mechanisms for NSCLP may differ among ethnic groups (Botstein and Risch 2003).

Variants in noncoding regions that modify transcription and gene expression have been increasingly implicated in complex disorders because they perturb transcription by creating or removing binding sites for specific transcription factors (Thesleff 1998; Wyszynski 2002a; Wang et al. 2005). In silico analyses suggested that rs138557689/C is found in a conserved H3K27Ac site and creates a novel protein-binding site that represses promoter activity of *FZD6* (Table S4). Moreover, we showed that both knockdown and overexpression of *fzd6* in zebrafish results in craniofacial defects (Fig.3).

**Figure 4:**
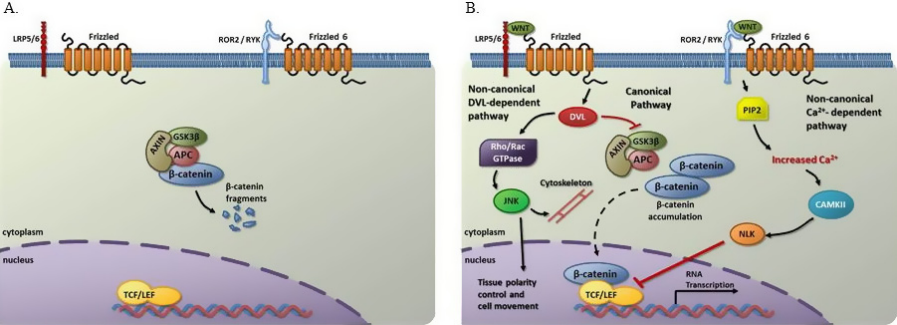
Schematic model of WNT signaling by the FZD receptor(s). (A) In absence of WNT, *β*-catenin is degraded by AXIN-APC-GSK3*β* complex. (B) Binding of WNT to FZD receptor (plus specific other coreceptors) results in transcription of WNT target genes by the canonical pathway, mediation of tissue polarity control by noncanonical DVL-dependent pathway, and inhibition of RNA transcription of WNT target genes by noncanonical Ca^2+^- dependent pathway. APC, adenomatous polyposis coli; AXIN, axis inhibition protein; CAMKII, Calcium/calmodulin-dependent protein kinase II; DVL, disheveled; GSK3*β,* glycogen synthase kinase 3 beta; JNK, Jun *N*-terminal kinase; LRP5/6, low-density lipoprotein receptor-related protein 5/6; NLK, Nemo-like kinase; PIP2, Phosphatidylinositol-4,5-bisphosphate; Rho/Rac, Small GTP-binding proteins; TCF/LEF, T-cell factor/Lymphoid enhancer-binding factor; WNT, Wingless-type MMTV integration site family.

*FZD6* is part of the *Frizzled* family of genes that encode a group of G-coupled receptors critical for initiation of WNT signaling and is expressed in the mandible and maxilla during murine development (Tokuhara et al. 1998; Borello et al. 1999; MacDonald et al. 2009). WNT signaling is a highly controlled cellular pathway that regulates multiple functions during craniofacial development through both noncanonical and canonical/*β*-catenin signal transduction (Fig.4) (Logan and Nusse 2004; Moon et al. 2004; De Calisto et al. 2005; Jiang et al. 2006; Brugmann et al. 2007; Song et al. 2009; Mani et al. 2010; Mazemondet et al. 2011; Reid et al. 2011; Wang et al. 2011). It has been suggested that *FZD6* represses canonical WNT signaling through the noncanonical Ca^2+^/CaMKII pathway by downregulating TCF/LEF-binding activity and subsequent transcription of WNT target genes (Fig.4) (Golan et al. 2004). Therefore, we hypothesize that decreased *FZD6* expression could lead to dysregulation of the WNT signaling pathway, a tissue-dependent effect leading to isolated clefting. Support for this theory comes from studies showing severe craniofacial abnormalities in mice deficient for *Dkk1* (Dickkopf-related protein 1**),** which is also a negative regulator of the WNT pathway (Mukhopadhyay et al. 2001). Additionally, a long-range murine enhancer of *Myc* expression causes dysregulation of *Fzd6* as well as *Wnt5A, Wnt9b, TCF4, Dkk1, and Lef1*, all WNT pathway genes (Uslu et al. 2014). This suggests that any alteration in physiological levels of fzd6 results in abnormal craniofacial development and that fzd6 levels must be finely regulated for normal development to occur. Altogether, these findings suggest a complex role for this WNT regulator in development.

*FZD6* also mediates the noncanonical planar cell polarity (WNT/PCP) pathway, which controls the polarity and orientation of the migrating neural crest cells (NCCs)(De Calisto et al. 2005; Matthews et al. 2008) (Guo et al. 2004; Wang et al. 2006; Devenport and Fuchs 2008). During normal craniofacial development, NCCs migrate from the neural folds to fill the facial prominences with mesenchyme and thus, contribute to the formation of the nose and upper lip (Senders et al. 2003; Chai and Maxson 2006; Jiang et al. 2006; Moore and Persaud 2008; Cordero et al. 2011). Defects in NCC formation, induction, differentiation or migration can result in craniofacial abnormalities (Garg et al. 2001; Vitelli et al. 2002; Dixon et al. 2006; Jones et al. 2008). Therefore, decreased *FZD6* expression could perturb the WNT/PCP pathway and alter NCC migration or mesenchymal planar cell polarity in craniofacial structures, potentially causing craniofacial anomalies including NSCLP. Our studies of zebrafish *fzd6* are in agreement with a possible disruption in NCC migration or planar cell polarity specification.

Intriguingly, while *Fzd6* null mice have abnormal or absent claws, as well as defects in hair patterning, they do not express orofacial clefting (Guo et al. 2004; Frojmark et al. 2011). This phenomenon may be similar to that in other human NSCLP genes such as *TGFΑ*, in which knockout mice do not have a cleft and wherein *TGFΑ* is thought to function as a modifier (Miettinen et al. 1999). Similarly, a spontaneous recessive hypomorphic mutation of *Wnt9b*, called *clf1*, is present in the A/WySn mouse strain and other closely related strains (Juriloff et al. 2004, 2005, 2006). About 5–30% of these strains have isolated CLP at birth(Juriloff et al. 2004). Penetrance of CLP ranges between 10% and 90% depending on genotype of the modifier gene, *clf2*. Furthermore, *Wnt9b2/clf2* mutant mice also display incomplete penetrance of CLP(Juriloff et al. 2006). Similarly, *FZD6* may act as a modifier gene to increase the risk of NSCLP in a susceptible genetic background. Interestingly, our *fzd6* overexpression phenotype (Fig.3F) mimics that of the zebrafish *silberblick* mutant line, which has a loss of function mutation in the zebrafish *wnt11* gene (Heisenberg et al. 1996, 2000). This suggests that *fzd6* overexpression interferes with *wnt11* function. Additional studies are needed to further examine the role of *fzd6* in craniofacial development. Taken together, our results complement previous studies and strongly implicate the FZD6/WNT signaling pathways in NSCLP (Juriloff et al. 2006; Chiquet et al. 2008; Geetha-Loganathan et al. 2009; Menezes et al. 2010; Yao et al. 2011; Letra et al. 2012; Mostowska et al. 2012; Kurosaka et al. 2014). Future studies in NSCLP should focus on interrogation of the genes in this pathway including regulatory elements using next-generation sequencing technology to capture of all the genetic variation in order to identify at-risk genotypes.

## Supporting Information

**Figure S1.** Linkage region 8q21.3-24.12 contained several candidate genes. Schematic depicting where in the linkage region genes of interest are located. **Figure S2**. *FZD6* rs138557689/A is conserved across species. UCSC Genome Browser Multiz Alignments of the human *FZD6* gene and homologs from other species. Arrow indicates the position of rs138557689 variant. The A allele is conserved across six species; mouse, rat, *X. tropicalis* and zebrafish lack the region containing this variant.

**Table S1.** *FZD6* primers sequences. **Table S2.** *MATN2* primers sequences. **Table S3.** *SLC25A32* primers sequences. **Table S4.** In silico results of rs138557689.
