# Supplementary material for: Regulatory variant in FZD6 gene contributes to nonsyndromic cleft lip and palate in an African-American family
Source: Mol Genet Genomic Med. 2015 May 7;3(5):440–51. doi: 10.1002/mgg3.155 (PMC4585452; doi:10.1002/mgg3.155)
Supplement: Supplementary file 1 [file mgg30003-0440-sd1.docx]

**Supplemental Figure Legends**

**Supplemental Figure 1. Linkage region 8q21.3-24.12 contained several candidate genes.** Schematic depicting where in the linkage region genes of interest are located.

**Supplemental Figure 2**. ***FZD6*** **rs138557689/A is conserved across species.** UCSC Genome Browser Multiz Alignments of the human *FZD6* gene and homologues from other species. Arrow indicates the position of rs138557689 variant. The A allele is conserved across six species; mouse, rat, *X. tropicalis* and zebrafish lack the region containing this variant.

**Supplemental Figure 1. Linkage region 8q21.3-24.12 contained several candidate genes.**

**
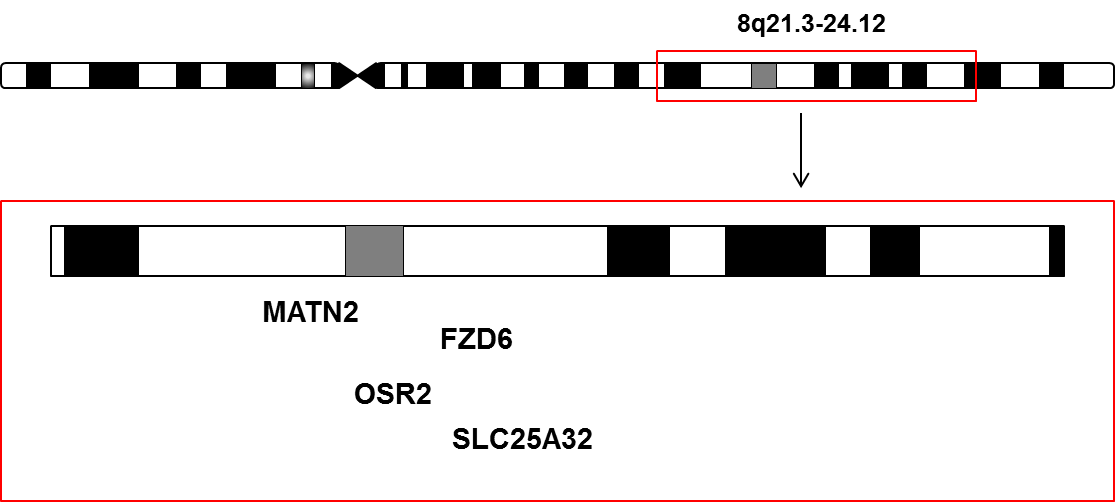
**

**Supplemental Figure 2**. ***FZD6*** **rs138557689/A is conserved across species.**

**
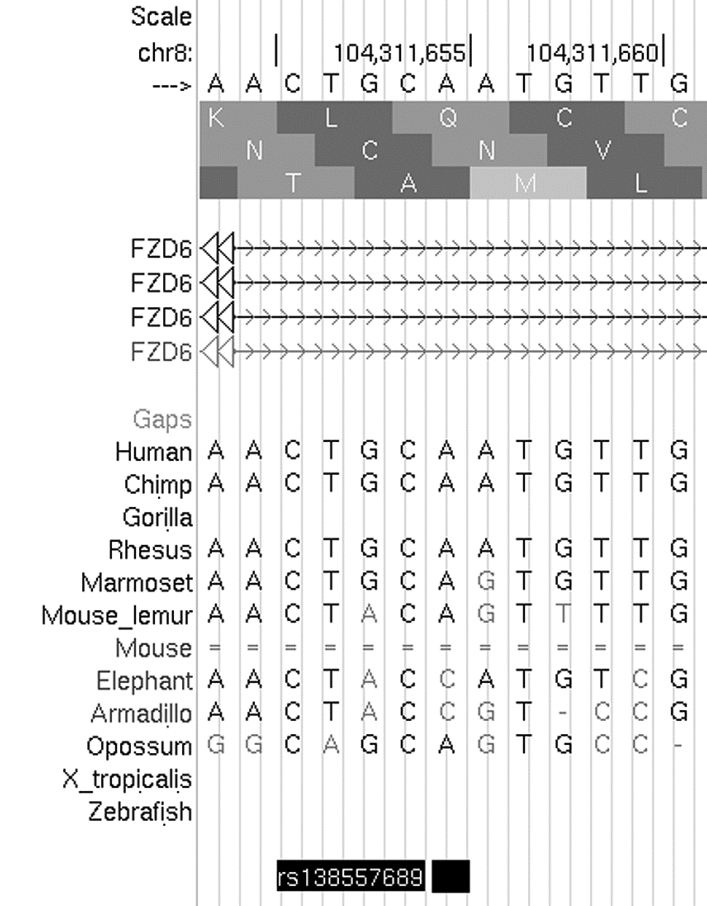
**
