# Supplementary material for: Regulatory variant in FZD6 gene contributes to nonsyndromic cleft lip and palate in an African-American family
Source: Mol Genet Genomic Med. 2015 May 7;3(5):440–51. doi: 10.1002/mgg3.155 (PMC4585452; doi:10.1002/mgg3.155)

**Supplemental Tables**

Supplemental Table 1. *FZD6* primers sequences

Supplemental Table 2. *MATN2* primers sequences

Supplemental Table 3. *SLC25A32* primers sequences

Supplemental Table 4. *In silico* results of rs138557689

**Supplemental Table 1. *FZD6* primer sequences**

| **Name** | **Forward** | **Reverse** | **Length (bp)** | **Tm(⁰C)** |
| --- | --- | --- | --- | --- |
| E1A | CACTGCTACCTGAGCATCCA | GGA AGC CAC CTC CAC CTT | 318 | 65.1 |
| E1B | AGA GCC AGC GCC AAG AGC TTC A | AGG GTG TAG TGC TGC CGT CGA AA | 874 | 65.1 |
| E1C | GGG AAC CGG CTC TGA AAG GCG | CCT TAA TAA GCT TTC CAA CAG GGG CCC | 466 | 69.4 |
| E1D | CTA GTT GGC CTT ACG AAA ATC GAG | GAC CCA GGA CTC ATT TTC AGG | 514 | 60.2 |
| E2 | CAAATGTTGCTGATACACCCTC | CACAACTTGAAGAAATCGGCTC | 473 | 54.3 |
| E3 | GAGTTCATAAGTCTGATAGAGGG | CTGTAAGTTCCCTGAGAGCAAG | 544 | 54.3 |
| E4A | CCCCATTAACAGCCACAAGTTTT | CGCCTAGCAAAAATCCAATGAAG | 593 | 54.3 |
| E4B | GCAACTCTGTTCACATTCCTTAC | GTACAAAGTAGCGAGAAGCATCC | 481 | 54.3 |
| E4C | GCAGTGTGGTTTCATGCTGTTG | GGCTCTTGTATTTTCTCACCTG | 538 | 54.3 |
| E5 | GATAAAAAATGTGTTGCACTTAGAGC | GATAAAAAATGTGTTGCACTTAGAGC | 486 | 54.3 |
| E6A | CTGACAAAAGCAACTTAGAGTG | CCTCTCTCATTGATGTTTCTGG | 472 | 54.3 |
| E6C | GGTCATTTCCAAATCCATGGG | CAAACTTCCTTGGGGTAAGAG | 427 | 54.3 |
| E7A | GATAAAGGTGGACACTGGTTAG | GAAGATTCCTCTAACTCTGTCC | 530 | 54.3 |
| E7B | GCATTGCCTACTGTTATACTGG | GGACACTCTTTGAGTAAGCACC | 592 | 54.3 |
| E7C | CCCACTTATTGATACCTTACCATC | CAAAAGCATCAGAAAATCTTGCCC | 576 | 54.3 |
| E7D | GCCAATCAAATGGAAAAAAGGTAG | GCATTCTCCTTCAAGGTTAAAAAG | 450 | 54.3 |

E=Exon

**Supplemental Table 2.** ***MATN2* primer sequences**

| **Name** | **Forward** | **Reverse** | **Length (bp)** | **Tm(⁰C)** |
| --- | --- | --- | --- | --- |
| E1A | GCT ACT CTG AGG CAG GAG AAT GG | GGC TAA TTG ATG ACA AGC GCC AAG | 572 | 63.5 |
| E1B | GAC TTT CCC TGC TCC CTC GGG GTA GG | C CGG GCG GAA GGA GGG GTG TCC | 538 | 65.1 |
| E1C | GGGAGCGCTCTGGGATGGGAC | CCT GTC CTC GGA GGG GTC GAA G | 374 | 60.2 |
| E1D | CAT CCCCGC GTC AGT GGG TGC | GAG GAC GGA GCC CCA GCT TCT | 499 | 63.1 |
| E2 | CGGCATTTTGAGTGCAAGTGG | CCAGCACATCCAACAAGGAC | 417 | 63.5 |
| E3A | GTAGAGACAGGGTTTTACAGTG | CCTCTGCTTCTGAGAATGCT | 465 | 63.5 |
| E3B | CGTGCTGTCAAGAGGATGCG | CCTATTGCCAGCACAGTACCTG | 459 | 63.5 |
| E4 | CTGCCCAGAGGAGAGAGTAG | GGGATGATAACTGGGGTGGG | 342 | 60.2 |
| E5 | GTTACTTTGGTGAGGGCTCTG | CTGAGCACTTTGTGGAAACCC | 352 | 63.5 |
| E6 | GCCCTCATCCTACCATTCCC | GTTGAGATACAGTGGTCTATGGTC | 383 | 63.5 |
| E7 | CCCCTTCATGGTGTGACTC | GCTTAAGATTTGCACGGAAAGG | 338 | 60.2 |
| E8 | GAATGATCCGTCCCGGCTTG | GACCATCAGGAAACCCGGTG | 375 | 63.5 |
| E9 | GACACCTTCCCTGTGGCTTG | GACACCTTCCCTGTGGCTTG | 305 | 63.5 |
| E10 | GCATGCCTTCGAGGGAGGGC | GGCCCAGCAAAGCCTGGAGAC | 315 | 63.5 |
| E11 | GGCCCAGCAAAGCCTGGAGAC | CCATTTTCTCTTTCAGGGTCC | 400 | 60.2 |
| E12 | CCATGGACCACTGAGCTCAGG | GAGCACCCAAGGCAGGAACTG | 379 | 63.5 |
| E13 | CCCCATCCTGAGTATGAGAC | GCAGTTAAGCCCTCTCTTCC | 440 | 63.5 |
| E14 | GGTTCCCCAAAGTGGTTATGCC | CTGTACACTCAAAGAAGCTTACCC | 625 | 63.5 |
| E15 | GTTGGCATGGACTCTTCAAATC | GCTGAAGCATGAGAATCACTTG | 446 | 63.5 |
| E16 | GCTTTTTTGAATCTTTGGTGTTACC | GTAAATGTTAACTATCTTTTTGAGGG | 372 | 63.5 |
| E17 | CCAAGTGCTAGGAATACCAAG | CTATTTCCTACTTCCTTCTTTCC | 338 | 56.2 |
| E18 | GGTATTTACTGGATCTGGCTGC | CTTGCCCTTGCCTCTCAAAC | 425 | 63.5 |
| E19A | GAG TTC TAC AAA TTT ACA AGT CAG GG | CA AAT GAG ATT GCA CAC TAA GGC | 542 | 56.2 |
| E19B | GTGAGAATGAATAAGCTATGCAAG | CCACATCCTTACCAACACTTG | 562 | 62 |
| E19C | GAG AAA TGG CCA ACA TGC CTA TGA AAA AAA TGC TG | CTA CAG GCA CCC ACT ACC ACA CCC G | 595 | 68.2 |

E=Exon

**Supplemental Table 3. *SLC25A32* primer sequences**

| **Name** | **Forward** | **Reverse** | **Length (bp)** | **Tm(⁰C)** |
| --- | --- | --- | --- | --- |
| E1A | CAT CTC GGT TGC TCT TCC GGC | CGC CCC TTG TGA GCG CAA C | 431 | 67 |
| E1B | CCC CTC CAT CGC GCT TTC CG | CAG GTT AGC CAA CGC GGA CAG | 513 | 67 |
| E2 | CGG GAC TCT GAC ACA AAA ATG | GCC ATT TTG TTC TTC CTG ATC C | 519 | 62 |
| E3 | GCA AAC CAC TTC CAG CAA ATT C | CTG AGG CAG GAT TAT CGC TTG | 530 | 58.3 |
| E4 | CCT TTT GAC CCT AAG ACT GTG C | GAT ACA TCC TAT GTT AAT GGG GAC | 537 | 58.3 |
| E5 | GTG CTG TAA TGA GAG AGA AGA G | GAC ATT TGT GTG GCT TCA | 374 | 58.3 |
| E6 | GAT GAG TAC TGG CTC TGC CA | CGT AAT AAC TGG GAA AGC AGG | 473 | 58.3 |
| E7A | GAC TGC TGC TTG CTC CAT GCC | GGC AGC CAT TTC AGG CAG AGG | 454 | 58.3 |
| E7B | GAA GCC AGA GAA CTG CTA AGT C | CGA CAA AGC AAG ACT CCA TCT C | 404 | 62 |
| E7C | GTG AGC TTA CTT GCC TGG ATT GC | CGC TAG GTA GTG CAT CCC AAC TG | 543 | 62 |
| E7D | GGATGGTCTCAATCTCCTGAC | CAA CCT GAA TTT GAG AAA CCA ATG AAG | 564 | 58.3 |
| E7E | GTTGACAAGGTAAATGGAAATGAG | GTT CAC TTT TTC CGT GTG GGG | 517 | 58.3 |
| E7F | GCT GTT GCA CTA CCA TCT ATT TG | CTC AGT GCT TGG TGA CGT AC | 556 | 52 |

E=Exon

**Supplemental Table 4. *OSR2* primer sequences**

| **Name** | **Forward** | **Reverse** | **Length (bp)** | **Tm(⁰C)** |
| --- | --- | --- | --- | --- |
| A | GAAATTTCTCCTGGGGCATT | GGGAGTCTTGGGCTTTAGTAGC | 721 | 63 |
| B | GAAGCAGTTGTTGAGTTTCTAATGG | CCAAGGAAGTGAGAAATACAGTGC | 852 | 51.1 |
| C | ACTCTGACGAAGACGTGGATCT | CCTCCACAAACACCTTTTCCT | 826 | 57 |
| D | CTGGTCTACGTACACTGAGATCAAA | GAGTTTCGCTCTTTGGTAATGG | 884 | 55 |
| E | AACAGAACCTAGATTATGCGGAAG | ACCTTAAATATCCACCAATGGCTCT | 836 | 62 |
| F | GACTGATGAAAGAAAAGTAGCCTCA | GCGTAATGAGGATTTTCCAGAGAT | 815 | 60 |
| G | ACCATTCAGAGAAAAGGGAAAGAAG | CATTTGAACCCAGCAGGAAG | 832 | 60 |
| H | GATTACTAATGGGGGTTTGCAGT | AACCCCTTTTCCCAGACTGA | 707 | 64 |
| I | GCTGTGAATGTAGGTGAGGTGAT | CTGTTAGGCGAAGCTCAGTCA | 711 | 63 |
| J | CTGAGGTGTCACTTTTCTTTCCTG | AGAGCCTTGCTCCCCATTTT | 860 | 50.2 |
| K | CAGTGGAGCACTTCTTGTTCTG | CCGCAAAACTTGCAGATAAACT | 861 | 64 |
| L | GTATCCCAATGTGCACGAGAT | GGACGAAACCCAACAGAAGTAG | 857 | 58.3 |
| M | GAGAGGGAAAGCGAATTTGTC | CTTTATCTACACTTGATCGCCCTCT | 1204 | 63.5 |
| N | CGCGACCTTAAACTCTGCATTA | TAAATGCAGGACAGATTGGATG | 864 | 62 |
| O | ACCATGGGGCAAAGTTATTTC | GCACCCTTTTCAATAGTTTCTGTCT | 839 | 62 |
| P | TAGCTTTCAGGACATTGAGGAATAG | CAGAGCTCTAAAATGAGCTGTGAAT | 394 | 62 |
| Q | AGTGTTCAGGCGAAACTGTGAT | TATCTAAGTGCATCCAACAAGATCC | 721 | 63 |

**Supplemental Table 4. *In silico* transcription binding sites**


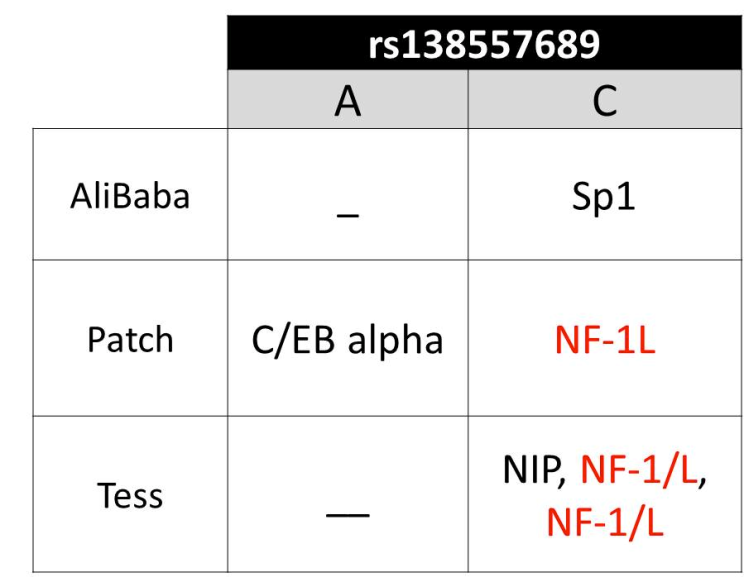

Supplement: Supplementary file 2 [file mgg30003-0440-sd2.docx]
